# Building a Competency Framework to Integrate Inter-disciplinary Precision Medicine Capabilities into the Medical Technology and Pharmaceutical Industry

**DOI:** 10.1007/s43441-024-00626-5

**Published:** 2024-03-15

**Authors:** Nicholette Conway, Orin Chisholm

**Affiliations:** 1 GenomePlus Pty Ltd, Sydney, Australia; 2 Sydney Pharmacy School, Faculty of Medicine and Health, The University of Sydney, Sydney, NSW 2006 Australia

**Keywords:** Precision medicine, Industry, Competency, Capabilities, Workforce, Interdisciplinary

## Abstract

**Introduction:**

Integration of precision medicine (PM) competencies across the Medical Technology and Pharmaceutical industry is critical to enable industry professionals to understand and develop the skills needed to navigate the opportunities arising from rapid scientific and technological innovation in PM. Our objective was to identify the key competency domains required by industry professionals to enable them to upskill themselves in PM-related aspects of their roles.

**Methods:**

A desktop research review of current literature, curriculum, and healthcare trends identified a core set of domains and subdomains related to PM competencies that were consistent across multiple disciplines and competency frameworks. A survey was used to confirm the applicability of these domains to the cross-functional and multi-disciplinary work practices of industry professionals. Companies were requested to trial the domains to determine their relevance in practice and feedback was obtained.

**Results:**

Four PM-relevant domains were identified from the literature review: medical science and technology; translational and clinical application; governance and regulation and professional practice. Survey results refined these domains, and case studies within companies confirmed the potential for this framework to be used as an adjunct to current role specific competency frameworks to provide a specific focus on needed PM capabilities.

**Conclusion:**

The framework was well accepted by local industry as a supplement to role specific competency frameworks to provide a structure on how to integrate new and evolving technologies into their current workforce development planning and build a continuous learning and cross-disciplinary mindset.

**Supplementary Information:**

The online version contains supplementary material available at 10.1007/s43441-024-00626-5.

## Introduction

Precision medicine (PM) represents a personalized approach to medical treatment that leverages an individual’s genetic profile and utilizes data and genomics to deliver tailored interventions [1, 2]. The evolution of PM has prompted growth of diagnostic and prognostic capabilities such as the identification of biomarkers for early disease detection and monitoring of treatment responses, and their utilization as companion diagnostic tools for determining optimal therapeutic interventions [3–5].

The Medical Technology and Pharmaceutical (MTP) industry workforce is critical to the translation of new technologies from research and development through pre-clinical and clinical phases of development as they build clinical, quality, and economic evidence required for clinical and commercial application of these therapies [6, 7]. The development and implementation of therapeutics into the health system requires a workforce that includes roles ranging from clinical research, data management, health economics, regulatory affairs, medical affairs, manufacturing, sales and marketing to roles that include new and evolving OMIC and data science technologies. Some of these roles are governed by role specific competency frameworks, internal training and formal capability management however, many of these do not provide a focus on competencies around PM [8–13]. The central challenges to integrating PM capabilities into the MTP workforce include the varied educational and academic backgrounds of staff across different industry roles and the lack of consistency with respect to PM capabilities required across different industry roles.

Competencies are fundamental characteristics that an individual requires for effective job performance, while competence relates to the job requirements that must be met to complete a specific task [14, 15]. An organizing framework that outlines the competencies necessary for job performance is called a competency model or framework. There are different approaches to developing such frameworks, with the most common being the identification of a set of “core competencies” along with specific competencies for different subgroups [14].

Competency frameworks provide important ‘infrastructure’ to enable organisations to build workforce capability. They:

- define the knowledge, skills and attitudes required for professional practice
- complement existing individual knowledge, skills, and attitudes and
- provide a standardised and consistent basis for tailored learning.

Internationally, there has been significant development on defining competencies for Health Care Providers (HCP), nurses, genetic counsellors and pharmacists with respect to PM, such as those developed by Genomics Education Resource Centre’s Genetics/Genomics Competency Centre (G2C2), while the UK’s Genomic Education Programme, has identified competencies based on tasks however, there are no such competency frameworks for professionals working in the MTP industry [16–21].

This paper describes the development and application of a PM competency framework for the MTP industry that can be used in addition to a role specific competency framework, and act as a guide to the development of the technical skills and cross-functional capabilities required to translate new PM-based discoveries into clinical practice. The purpose of this framework is to provide a structure to education and training activities and support the development of an MTP industry workforce that is:

- confident and capable of integrating genomic information and/or technologies into their everyday work practice and
- foster a culture of continuous learning to deliver genomic scientific, technology and healthcare integration and innovations across the MTP industry.

## Methods

### Literature Search

**Figure 1:**
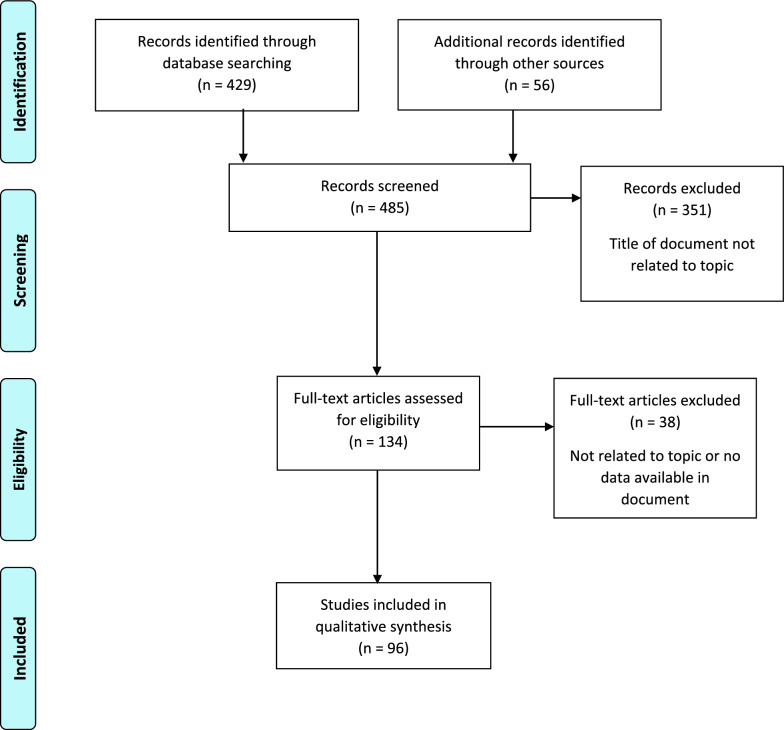
Search strategy.

A comprehensive literature search of the PubMed database was conducted in August 2021, with no restrictions on the publication dates, study design or country of origin, although we did restrict our search to publications written in English. Various search terms were used including: “precision medicine workforce capability”, “precision medicine workforce competenc*”, “workforce development in precision medicine”, “precision medicine training” AND “pharmaceutical industry OR “medical device industry”, “genomics training”, “genomics competenc*”, as listed in the search results (Table 1). A subset of potentially relevant articles was obtained for further review based on article titles, and a snowball technique was utilized to identify additional relevant references. One investigator conducted an initial review of the publications for inclusion, and relevant articles were subsequently reviewed by the other investigator. The search strategy is outlined in Fig. 1.

**Table 1:** Search terms and number of publications obtained

Search term	Number of articles obtained	Number of potentially relevant articles retrieved after title review	Number of publications included in development of framework
Precision medicine workforce capability	4	3	1
Precision medicine workforce competencies/ workforce development in precision medicine	75	36	20
Precision medicine training pharmaceutical industry	65	3	3
Precision medicine training medical device industry	23	0	0
Genomics training medical device industry	108	0	0
Genomic competencies medical device industry	96	0	0
Genomic(s) competencies	38	17	14
Genomics training	13	13	7
Competency medical devices or Biomedical engineering competency framework	6	6	5
Precision medicine and diagnostics and competency	1	1	1
Publications from snowball	29	29	23
White papers	10	10	6
Higher education curricula reviewed	17	16	16

In addition, white papers from recognized organizations that are involved directly in PM or in analysing its impact on the healthcare environment were targeted for inclusion in the analysis since these documents could discuss skills, knowledge and attributes required for a PM workforce. Finally, targeted searches were performed using the Google search engine to identify PM educational programs offered by various higher education institutions. These were reviewed for curricula since this could inform competencies identified as relevant to PM. Full search results are available on request.

### Synthesis of Information for Draft Competency Framework

Results of the literature review and current higher education curricula was used to identify a consistent set of domains and subdomains as being relevant to multiple cross-disciplinary stakeholders across the MTP sector. Competency frameworks for specific industry roles were also reviewed for the most appropriate structure to use in developing the PM competency framework.

### Survey

To assess the relevance of the domains and sub-domains identified to current and future PM applications, the draft framework was presented to a cross-functional group of professionals from across the MTP industry, along with a set of questions in an online survey, designed using SurveyMonkey. A group of Industry Genomics Network Alliance (InGeNA) member company representatives provided feedback on the survey questions. InGeNA members were considered representative of the appropriate industry audience as all members were currently working in an industry role in PM, and the companies or organisations they represented were active in various stages of product development across the MTP industry. The survey link was distributed to representatives of InGeNA to distribute within their companies. The survey was open for one month during September–October 2021 and contained a consent page before participants could enter the questionnaire. It included 25 questions covering participants’ demographics, their perceptions of their individual experience and future training needs in precision medicine, and their perceptions of the draft framework and competency requirements for their current roles and those same roles in 5 years’ time. The full set of survey questions are provided in Appendix 1.

### Consolidation of Framework

Following analysis of the survey results, and further discussion with InGeNA representatives, domains were consolidated, and the PM competency framework was finalised.

### Pilot Testing of Framework

Two companies agreed to pilot test the framework in their workplace and provided feedback on the applicability of the framework in their context.

### Release of the Framework

The framework is publicly available on the InGeNA website [22].

## Results

### Literature and Curricula Review

From 485 references, curricula documents and white papers obtained, 134 were identified as potentially relevant and 96 were included in the final review and contributed to the development of the framework.

The 96 documents included 16 post-graduate curricula and 80 additional documents, the majority of which focused on the United States of America (55%) and the United Kingdom or European Union (21%). Fewer documents related to global (13%) or the Australia/Asia–Pacific region (6%). These documents had varying degrees of relevance to different healthcare professions, with allied healthcare professionals (29%) and clinical medicine and pharmaceutical industry professionals (24% each) being the most common focus areas. About 18% of the documents referred to the general healthcare system, and there were five documents referring to the medical devices industry.

The specific competency frameworks identified for PM or genomics were well advanced for the nursing profession, followed by frameworks for other healthcare providers such as physicians, genetic counsellors and pharmacists. The competency frameworks developed for these professions included domains around integrating genomics into patient care and communicating complex information to patients. All the frameworks included basic genetic concepts as a critical domain, and ethics, legal and societal impact were heavily emphasised across several frameworks. All had some element of professionalism/professional practice included in them.

Concepts identified from the literature as important in PM included: machine learning (ML), mathematical modelling, computation, bioinformatics, big data, data visualisation, management and safety, the need to be “problem-solvers” and able to collaborate across disciplines [7, 23–26]; a need to understand the clinical trial enterprise and its governance and an ability to integrate molecular information, as well as an understanding of the technologies involved and being able to manage complex data [27, 28]. Competencies required by pharmacists included basic genetic concepts, genetics and disease, pharmacogenetics/pharmacogenomics, ethical, legal and social implications and an ability to communicate complex concepts [21, 29, 30]. Many of the papers discussed PM competencies required by nurses including understanding new clinical trial designs, genomics, cell and molecular biology, bioinformatics, understanding of the utility and limitations of genetic/genomic information, culturally appropriate communication skills, ethics, legal and social issues surrounding PM, professional responsibility and leadership [31–37]. Competencies identified for healthcare practitioners included interpersonal and communication skills, interprofessional collaboration, delivering appropriate treatment based on genomic results, problem-solving skills, data analysis and interpretation, being able to connect molecular targets and disease pathogenesis, understanding of omics, data analytics, legal, ethical and social aspects of pharmacogenomics, informatics, impact on patients, critical thinking skills, lifelong learning, and systems-based practice [20, 38–41].

Industry-specific roles also had several relevant frameworks, which informed the development of the draft framework for PM [8–13]. The engineering competency frameworks and medical device regulator frameworks included specific domains around engineering skills, and professional and personal attributes [42–47]. These frameworks had specific domains around their areas of expertise but also had domains that were relevant across the industry, such as ethics, leadership, professional practice, twenty-first century skills and business skills. Of note, Hartl et al. [6] addressed the drug development perspective of PM and identified multi-omics profiling, biomarker-guided trial designs, model-based data integration, artificial intelligence (AI), digital biomarkers and patient engagement as critical areas for the future of drug development. These aspects are critical in the basic science competencies for PM.

A comprehensive overview of higher education programs related to PM or genomics identified seventeen higher education curricula for master’s-level degrees, with four programs from North America, five from Australia, and eight from the UK [48–64]. Notably, the UK appears to have a more advanced development of relevant master’s-level education programs in PM, which could be attributed to the oversight of genomics education by the National Health Service (NHS) through their genomics education program [65, 66]. This program has prioritized genomics education in the UK healthcare system and has collaborated with the higher education sector to provide relevant programs.

A review of the curricula covered in these degrees provided valuable insights into the competencies that educators perceive as important in the development of precision medicine skills. The UK-based courses were specifically designed to address the training needs identified by the NHS, including the fundamentals of human genetics and genomics, omics technologies, bioinformatics, ethical, legal, and social issues in genomics, and professional practice. Most of the Australian-based degrees also covered these areas, with a greater emphasis on basic and clinical genomics. The North American degrees primarily covered PM basics, clinical applications, and ethics and legal issues.

Based on the above literature review a draft competency framework was compiled that would be complementary to the competency requirements for cross-disciplinary MTP industry roles. The draft framework covered four main domains: medical science and technologies, translation and clinical application, governance and regulation and professional practice. Each domain contained several subdomains which had been identified from the literature search.

### Survey Results

The draft framework was evaluated to determine if there were other competencies that might be required to ensure the framework was fit for purpose and relevant for future use. An online survey was conducted among the eleven member companies of InGeNA and it was open for four weeks. InGeNA member companies were specifically targeted due to their self-selection as companies operating in the PM space within Australia. The survey received a total of thirty-three responses from the participants, all of whom provided their consent to participate in the survey.

#### Study Participants

The study participants possessed varying qualifications, industry experience, and years of expertise, thereby contributing to a diverse knowledge of PM. Of the 33 respondents, 67% held either a masters’ or PhD level qualification, while 27% possessed a bachelor’s or honour’s degree. Most respondents obtained a degree in a biological discipline, such as genetics, molecular biology, or pharmacy, whereas nine individuals held degrees in other areas such as business, finance, or economics/commerce. Participants held various roles within their companies, with the highest number identifying as working in sales or marketing roles (23%, 7/31 respondents). Other positions reported were in medical affairs or senior management (13% or 4/31 each), and business development (10%, 3/31). Most the respondents (81%;) had more than 10 years of experience working in the industry, with over half of the respondents (55%) employed in large global organizations (18/33 respondents). Most respondents were working in the pharmaceutical industry (61%; 20/33) or biotechnology industry (24%; 8/33), and a smaller proportion of respondents were employed in the diagnostics industry (18%; 6/33), and digital health (12%; 4/33). About half of the respondents reported working in the clinical development stage (48%, 16/33 respondents), followed by the sales and marketing stage of development (45%, 15/33), followed by pricing, reimbursement, and access stage of product development (39%, 13/33) and most respondents (61%, 20/33) claimed to possess either intermediate or advanced knowledge of PM.

#### Study Results

The survey participants confirmed the relevance of the domains and sub-domains to their current work and expected future work in 5 years’ time, to capture the evolving competencies required for PM in the future. There was strong acceptance of the four domains and with patient requirements specifically included for each domain. There was some variance between current requirements and predicted future requirements, and these variances led to the consolidation of some subdomains.

#### Medical Science and Technologies Domain

The results showed that 64% of the respondents believed they needed intermediate or advanced knowledge of diagnostic technologies and diagnostic testing, while the same percentage believed they needed this level of understanding in data science (Appendix 2). 55% considered that intermediate or advanced knowledge of genetics and genomics, digital technology, and risk analysis, efficacy, and effectiveness was necessary for their roles. However, the respondents gave less importance to basic cell biology, OMICS, biobanking, and AI and ML applications currently in product development. However, when asked to consider these skills for their roles in 5 years’ time there was a marked increase in the need to understand OMICS (increased from 39 to 88%), while a need to understand the other subdomains all increased.

#### Translation and Clinical Application Domain

A knowledge of patient diagnostic and treatment pathways was deemed essential for competency in current roles by most respondents (79%, 26/33; Appendix 2). Conversely, bioinformatics and data interpretation were not considered essential competencies for respondents’ current roles (39%, 13/33) but were considered necessary for their roles in 5 years’ time (85%, 28/33). Furthermore, there was a substantial increase in the perceived importance of clinical utility, levels of evidence, and genome analysis for respondents’ roles in 5 years’ time. Interestingly, genetic counselling was not deemed an important competency across the industry. However, respondents in sales and marketing and service provider roles in the biotechnology and diagnostics industries rated this competency highly, underscoring the variation in competency requirements across industries and roles.

Intermediate and advanced levels of knowledge across all competencies will be necessary in the next 5 years, particularly for patient diagnostic and treatment pathways, genome analysis and its implications, bioinformatics and data interpretation, and access and reimbursement. Some respondents also emphasized the need for deeper comprehension of the health economics principles that underlie Health Technology Assessment (HTA). As with previous sub-domains, there is a variation in competency ratings based on industry and roles.

#### Governance and Regulation Domain

The most significant competency required for both present and future roles is a comprehensive understanding of codes of conduct and compliance (88% (29/33) (Appendix 2)). The most significant change in competency requirements from current to future roles was observed for legal and regulatory requirements related to AI/ML (current: 27% (9/33); future: 81% (26/32)). Additional competencies with notable increases include legal and regulatory requirements for pharmaceuticals (from 61%, 20/33 to 88%, 29/33), legal and regulatory requirements for medical data (from 52%, 17/33 to 82%, 27/33), societal impacts and policy development (from 45%, 15/33 to 76%, 25/33), and legal and regulatory requirements for diagnostics (42%, 14/33 to 88%, 29/33).

#### Professional Practice Domain

Twenty-first century skills such as critical thinking, creativity, and problem-solving were deemed the most important competencies, with an increase in relevance from 61% (20/33) to 73% (24/33). Continuous learning and its impact on adaptability and innovation also showed a significant increase in relevance, from 30% (10/33) for current roles to 61% (20/33) for future roles.

#### Future Competencies and Training Needs

In a free text question, respondents provided additional comments regarding competencies and capabilities necessary for the translation of PM into the health system and the development of genomic technologies. The main concerns raised were the need for clear reimbursement pathways for PM and better training for health system employees to expand beyond their knowledge base. Respondents suggested additional competencies that would be required for industry training in the future, such as a demand for competencies in health economics, real world evidence, and reimbursement, as well as a better understanding of the overall regulatory requirements for PM. The majority of respondents expressed a need for further training in PM with short courses delivered by industry organizations being the most preferred method of training. The most sought-after training topics were related to data science, AI, and ML applications, followed by ethical and social implications, legal and regulatory aspects, and bioinformatics.

### The Final Framework

**Figure 2:**
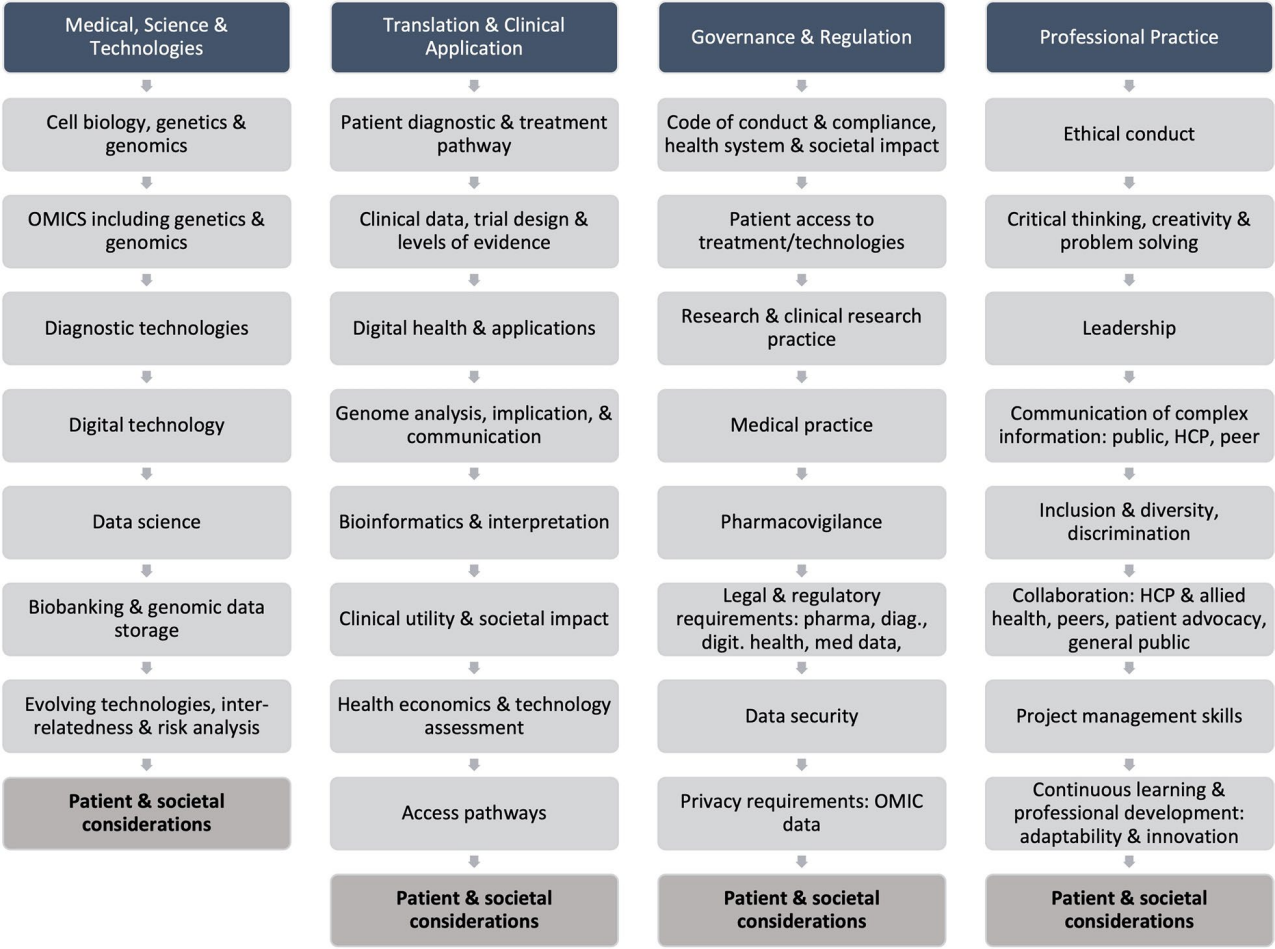
Precision medicine MTP industry competency framework.

Following the survey analysis and further discussions with industry experts the draft framework was updated. Given the rapid evolution of new technologies in healthcare and since the finalization of the framework, we have updated some of the descriptions used in digital health and data science to reflect current wording. This updated precision medicine MTP industry competency framework is outlined in Fig. 2.

### Pilot Case Studies

Several case studies were conducted by InGeNA members (personal communication). Below are examples from two case studies that used the PM framework to support the PM workforce needs.

#### Case Study 1: Strategic Project and Workforce Planning

Company A is an early-stage Australian company that has built a platform to accelerate the integration of genomic, digital and AI empowered personalised health information into the healthcare system. The team is made up of individuals with competencies across multiple areas including translational health and various clinical specialties, data science, AI, and genomics. The team members bring diverse research, clinical and industry experience, and the company needs to assemble the right team to deliver on the new technology solution. Traditional role-based workforce gap analysis does not work effectively in organisations working in evolving technologies where roles are cross-disciplinary and in many cases the role needs to be created. The PM framework provided a robust and fit-for-purpose scaffold to assess the interdisciplinary skills needed when planning for a new project and assess the capability required for each domain and subdomain.

Company A used the following approach:

- Used the PM domains to define the project scope and allocated the competency levels for subdomains needed to deliver on the project.
- Assigned team members with complementary skills and experience and identified the gaps in the overall skillsets.
- Addressed the gaps by insourcing specific skills and including some on-the-job training of existing personnel.

By knowing where to allocate resources such as training, recruitment and partnership development, innovative and lean companies, such as Company A, can be confident in creating a workforce that can deliver for future needs.

#### Case Study 2: Enabling Early Collaboration Between Biopharma and the Genomics Healthcare Ecosystem

Company B’s near-term pipeline includes gene therapies for diseases that have single-gene alterations, with the approach to develop highly specialised, potentially once-in-a-lifetime gene therapies that use custom-made vectors. The successful integration of clinical genomics, development of infrastructure, health system readiness and a highly skilled PM workforce are critical enablers of the success and speed in which these pipeline investigational agents can be translated from clinical research into therapies delivered within person-centred models of care.

The PM competency framework was used to contextualise these interdependencies and the taxonomy built into the framework allowed Company B to use a common language for genomics across stakeholders. By transcending these silos, Company B and other PM manufacturers have a much better opportunity to connect, gain early visibility, develop a common understanding and influence the changes required to existing systems, which are essential to realise the potential of these novel therapies.

Company B also stated that there is potential to leverage the competency framework to launch innovative partnerships such as preceptorships and skills transfer programs that seek to bridge the gap between biopharma and clinical research, which will benefit translational research and commercialisation opportunities and build a competent workforce. The company indicated that the PM framework allowed innovative approaches to recruitment and knowledge sharing.

These, and other case studies confirmed the validity and acceptance of the framework by industry. The framework has been made publicly available on the InGeNA website to enable any member of the MTP industry to use the framework in their professional development plans and discussions with their managers.

## Discussion

Key components of translational PM relevant to the pharmaceutical industry include multi-omics profiling, digital and molecular biomarkers, model-based data integration, AI, biomarker-guided trial designs, patient-centric companion diagnostics, and real-world evidence integration [6, 67]. Given this emphasis on PM, it is imperative that the workforce be continually educated and upskilled in this area and develops a mindset of continuous learning.

Our literature search revealed that current MTP industry competency frameworks tend to be role-specific, lacking reference to PM, innovation, and continuous or lifelong learning [8–13]. While some mention multi-sectoral collaboration or teamwork, all refer to professional standards, with some reflecting consideration of societal and ethical impacts [42–47]. The digital technology-focused frameworks are more comprehensive, reflecting their application across disciplines [68, 69]. We found that masters’ programs in this area usually focus on knowledge and technical competency [9, 70].

Given these findings, the MTP workforce needs a framework that includes critical aspects along with ethical principles, such as autonomy, beneficence, non-maleficence, and social justice. Any training material developed for industry must also consider the healthcare environment, as well as the governance, regulatory, and reimbursement structure, which are critical to collaborating across industries to integrate advancements. The framework presented in this paper is derived from several existing PM competency frameworks, incorporating professional capabilities and adjusted to meet industry needs based on feedback from InGeNA members from across the MTP industries in Australia and their diverse roles and experiences [14, 15, 71]. Emerging competencies in digital health and data science sub-domains as well as the inclusion of patient or person centric care are included [72, 73].

The twenty-first century competencies, combined with professional capabilities and a continuous learning mindset, are critical in developing the agility required in developing PM treatments and diagnostics for an evolving healthcare system [74–76].

The literature review and survey responses indicate a need for leadership in PM. Leadership can come from various levels within an organization, including senior leadership committed to PM principles and individual industry members demonstrating leadership through their work practices. The MTP industry requires a skilled workforce with key capabilities such as complex reasoning and adaptive thinking, agility and self-directed learning, and effective communication and persuasion skills [7].

The PM industry workforce competency framework was developed based on a review of current literature, curricula, and healthcare trends. The survey confirmed the relevance of the domains and subdomains identified in the desktop research, and confirmed the areas where competencies will need to be developed to support the implementation and development of PM by the MTP industry. The four domains identified include medical science and technology, translation and clinical application, governance and regulation, and professional practice. Each domain has a series of subdomains which cover capabilities required for the MTP industry with respect to genomics, data science and digital health and related technology developments. Patient needs and requirements should be considered across all four of the domains identified as relevant to the therapeutic area or specialty. The framework can be used by industry professionals, and their supervisors, to assess their development needs with respect to PM. It provides an opportunity for partnership on workforce education across industries, with patients and patient advocates as well as with regulators, government and payers to address competencies and skills needed to deliver new PM products.

### Supplementary Information

Below is the link to the electronic supplementary material.

Supplementary file1 (DOCX 31 KB)

Supplementary file2 (DOCX 51 KB)

## Data Availability

Complete survey responses are available upon request.
