# Supplementary material for: Building a Competency Framework to Integrate Inter-disciplinary Precision Medicine Capabilities into the Medical Technology and Pharmaceutical Industry
Source: Ther Innov Regul Sci. 2024 Mar 15;58(3):567–77. doi: 10.1007/s43441-024-00626-5 (PMC11043185; doi:10.1007/s43441-024-00626-5)
Supplement: Supplementary file 1 — Supplementary file1 (DOCX 31 KB) [file 43441_2024_626_MOESM1_ESM.docx]

| **Precision Medicine Competencies and Capabilities** |
| --- |

Consent

Welcome to the InGeNA Precision Medicine Competency Framework study!  

The purpose of this survey is to inform the precision medicine (PM) competencies and capabilities required for roles in the medical technology and pharmaceutical (MTP) industry to ensure we have an adequately trained workforce to meet future needs. You are requested to answer some questions about your demographics, your current understanding of precision medicine, and the competencies and capabilities required in your role and your training requirements for precision medicine. The initial set of questions will assist us in providing a snapshot of the understanding of Precision Medicine now by the MTP industry and the next set of questions aims to inform future training needs across the MTP industry.

Please be assured that your responses will be kept completely confidential. All surveys are voluntary and anonymous.

The survey should take you around 15 minutes to complete. If you would like to contact the Principal Investigators in the study to discuss this research, please e-mail Orin Chisholm at o.chisholm@pharmmed.com.au.

By clicking the button below, you acknowledge that your participation in the study is voluntary, you are 18 years of age or older, and that you are aware that you may choose to terminate your participation in the study at any time and for any reason and without any prejudice.

Please note that this survey will be best displayed on a laptop or desktop computer.  Some features may be less compatible for use on a mobile device. 

I agree and consent to this survey.

1. What is the highest level of education you have completed?

PhD

Fellowship of a medical college (e.g. RACGP or equivalent)

Medicine (either undergraduate or postgraduate)

Master's degree

Graduate Diploma

Graduate Certificate

Honours degree

Bachelor's degree

Post-secondary school certificate or diploma

Other (please specify)

2. Which of the following categories best describes your current employment status?

Employed, working full-time

Employed, working part-time

Not employed, looking for work

Employed, casual

3. How long have you been employed in the industry?

less than 1 year

1-4 years

5-9 years

10-19 years

20 years or over

4. In what type of setting are you employed? Please select all that apply.

Pharmaceutical industry

Biotechnology industry

Medical technology/diagnostics industry

Academic/basic research

Academic/clinical research

Healthcare setting (eg, medical practice, hospital)

Contract research organisation

Consultancy

Government

Other (please specify)

5. In your current role, what are the main functions you perform?

Research scientist/Engineer/Software developer

Pharmacologist/toxicologist/pre-clinical research and development

Biostatistician/data analyst/data scientist

Clinical research professional

Regulatory Affairs professional

Medical Affairs professional

Vigilance/safety specialist

Sales or marketing professional

Support functions (HR, finance, legal)

Health Technology Assessment/Health Economics

Manufacturing

Senior management/CEO/Managing Director

Consultant

Quality Assurance professional

Government/corporate affairs

Business development

Board member

Other (please specify)

6. What do you understand by the term "precision medicine"?

7. What type of training or education have you completed in precision medicine?

formal university degree

formal university subject

massive open online course (MOOC)

short course from an industry organisation (eg, ARCS, RAPS, DIA, etc courses)

free online course

courses provided internally by my company/organisation

my own reading

8. In which of the following areas of precision medicine would you like further training?

basic genetics and genomics

the role of genetics in disease

biomarkers

pharmacogenetics/pharmacogenomics

ethical, legal and social implications of precision medicine

bioinformatics

9. What size company do you work in?

Local company (less than 10 people)

Local company (10-100 people)

Local company (more than 100 people)

Global company (less than 1000 people)

Global company (1000-10,000 people)

Global company (more than 10,000 people)

10. For the role that you perform, at what stage of the development continuum is it?

Discovery research

Early-stage product development

Non-clinical development

Clinical development

Regulatory

HTA

Post-marketing product maintenance

Marketing and related support

Customer-facing role

11. For the category of "medical science and technologies" related to precision medicine, please rate your understanding of each of the following topics:

|  | no knowledge | basic knowledge | intermediate knowledge | advanced knowledge |
| --- | --- | --- | --- | --- |
| Cell biology - structure, function and behaviour of cells | Cell biology - structure, function and behaviour of cells no knowledge | Cell biology - structure, function and behaviour of cells basic knowledge | Cell biology - structure, function and behaviour of cells intermediate knowledge | Cell biology - structure, function and behaviour of cells advanced knowledge |
| Genetics - genes and their role in inheritance | Genetics - genes and their role in inheritance no knowledge | Genetics - genes and their role in inheritance basic knowledge | Genetics - genes and their role in inheritance intermediate knowledge | Genetics - genes and their role in inheritance advanced knowledge |
| Genomics - inter-relatedness of all of a person's genes | Genomics - inter-relatedness of all of a person's genes no knowledge | Genomics - inter-relatedness of all of a person's genes basic knowledge | Genomics - inter-relatedness of all of a person's genes intermediate knowledge | Genomics - inter-relatedness of all of a person's genes advanced knowledge |
| Diagnostic technologies and diagnostic testing - such as PCR, GWAS, FSH, NGS | Diagnostic technologies and diagnostic testing - such as PCR, GWAS, FSH, NGS no knowledge | Diagnostic technologies and diagnostic testing - such as PCR, GWAS, FSH, NGS basic knowledge | Diagnostic technologies and diagnostic testing - such as PCR, GWAS, FSH, NGS intermediate knowledge | Diagnostic technologies and diagnostic testing - such as PCR, GWAS, FSH, NGS advanced knowledge |
| Data science - scope, principles and use in healthcare | Data science - scope, principles and use in healthcare no knowledge | Data science - scope, principles and use in healthcare basic knowledge | Data science - scope, principles and use in healthcare intermediate knowledge | Data science - scope, principles and use in healthcare advanced knowledge |
| AI and machine learning and applications in product development | AI and machine learning and applications in product development no knowledge | AI and machine learning and applications in product development basic knowledge | AI and machine learning and applications in product development intermediate knowledge | AI and machine learning and applications in product development advanced knowledge |
| Biobanks and storage of genomic samples and data | Biobanks and storage of genomic samples and data no knowledge | Biobanks and storage of genomic samples and data basic knowledge | Biobanks and storage of genomic samples and data intermediate knowledge | Biobanks and storage of genomic samples and data advanced knowledge |
| OMICS technologies (genomics, proteomics, metabolomics, metagenomics, transcriptomics, etc) | OMICS technologies (genomics, proteomics, metabolomics, metagenomics, transcriptomics, etc) no knowledge | OMICS technologies (genomics, proteomics, metabolomics, metagenomics, transcriptomics, etc) basic knowledge | OMICS technologies (genomics, proteomics, metabolomics, metagenomics, transcriptomics, etc) intermediate knowledge | OMICS technologies (genomics, proteomics, metabolomics, metagenomics, transcriptomics, etc) advanced knowledge |
| Digital technology in healthcare | Digital technology in healthcare no knowledge | Digital technology in healthcare basic knowledge | Digital technology in healthcare intermediate knowledge | Digital technology in healthcare advanced knowledge |
| Risk analysis, efficacy and effectiveness | Risk analysis, efficacy and effectiveness no knowledge | Risk analysis, efficacy and effectiveness basic knowledge | Risk analysis, efficacy and effectiveness intermediate knowledge | Risk analysis, efficacy and effectiveness advanced knowledge |

12. For the category of "translation and clinical application" related to precision medicine, please rate your understanding of each of the following topics:

|  | no knowledge | basic knowledge | intermediate knowledge | advanced knowledge |
| --- | --- | --- | --- | --- |
| Levels of evidence – and how this may variation by clinical application | Levels of evidence – and how this may variation by clinical application no knowledge | Levels of evidence – and how this may variation by clinical application basic knowledge | Levels of evidence – and how this may variation by clinical application intermediate knowledge | Levels of evidence – and how this may variation by clinical application advanced knowledge |
| Clinical utility and societal impact – relevance and usefulness of an intervention | Clinical utility and societal impact – relevance and usefulness of an intervention no knowledge | Clinical utility and societal impact – relevance and usefulness of an intervention basic knowledge | Clinical utility and societal impact – relevance and usefulness of an intervention intermediate knowledge | Clinical utility and societal impact – relevance and usefulness of an intervention advanced knowledge |
| Genetic counselling and informed consent - purpose and process | Genetic counselling and informed consent - purpose and process no knowledge | Genetic counselling and informed consent - purpose and process basic knowledge | Genetic counselling and informed consent - purpose and process intermediate knowledge | Genetic counselling and informed consent - purpose and process advanced knowledge |
| Digital health and applications | Digital health and applications no knowledge | Digital health and applications basic knowledge | Digital health and applications intermediate knowledge | Digital health and applications advanced knowledge |
| Patient diagnostic and treatment pathways | Patient diagnostic and treatment pathways no knowledge | Patient diagnostic and treatment pathways basic knowledge | Patient diagnostic and treatment pathways intermediate knowledge | Patient diagnostic and treatment pathways advanced knowledge |
| Genome analysis and implications | Genome analysis and implications no knowledge | Genome analysis and implications basic knowledge | Genome analysis and implications intermediate knowledge | Genome analysis and implications advanced knowledge |
| Bioinformatics and data interpretation | Bioinformatics and data interpretation no knowledge | Bioinformatics and data interpretation basic knowledge | Bioinformatics and data interpretation intermediate knowledge | Bioinformatics and data interpretation advanced knowledge |
| Access and reimbursement - methodology and mechanisms | Access and reimbursement - methodology and mechanisms no knowledge | Access and reimbursement - methodology and mechanisms basic knowledge | Access and reimbursement - methodology and mechanisms intermediate knowledge | Access and reimbursement - methodology and mechanisms advanced knowledge |

13. For the category of "governance and regulation" related to precision medicine, please rate your understanding of each of the following topics:

|  | no knowledge | basic knowledge | intermediate knowledge | advanced knowledge |
| --- | --- | --- | --- | --- |
| Principles of research as applied to genomics | Principles of research as applied to genomics no knowledge | Principles of research as applied to genomics basic knowledge | Principles of research as applied to genomics intermediate knowledge | Principles of research as applied to genomics advanced knowledge |
| Medical practice | Medical practice no knowledge | Medical practice basic knowledge | Medical practice intermediate knowledge | Medical practice advanced knowledge |
| Vigilance as applied to genomics | Vigilance as applied to genomics no knowledge | Vigilance as applied to genomics basic knowledge | Vigilance as applied to genomics intermediate knowledge | Vigilance as applied to genomics advanced knowledge |
| Legal and regulatory requirements - pharmaceuticals | Legal and regulatory requirements - pharmaceuticals no knowledge | Legal and regulatory requirements - pharmaceuticals basic knowledge | Legal and regulatory requirements - pharmaceuticals intermediate knowledge | Legal and regulatory requirements - pharmaceuticals advanced knowledge |
| Legal and regulatory requirements - diagnostics | Legal and regulatory requirements - diagnostics no knowledge | Legal and regulatory requirements - diagnostics basic knowledge | Legal and regulatory requirements - diagnostics intermediate knowledge | Legal and regulatory requirements - diagnostics advanced knowledge |
| Legal and regulatory requirements - digital health | Legal and regulatory requirements - digital health no knowledge | Legal and regulatory requirements - digital health basic knowledge | Legal and regulatory requirements - digital health intermediate knowledge | Legal and regulatory requirements - digital health advanced knowledge |
| Legal and regulatory requirements - AI/ML | Legal and regulatory requirements - AI/ML no knowledge | Legal and regulatory requirements - AI/ML basic knowledge | Legal and regulatory requirements - AI/ML intermediate knowledge | Legal and regulatory requirements - AI/ML advanced knowledge |
| Legal and regulatory requirements - medical data | Legal and regulatory requirements - medical data no knowledge | Legal and regulatory requirements - medical data basic knowledge | Legal and regulatory requirements - medical data intermediate knowledge | Legal and regulatory requirements - medical data advanced knowledge |
| Legal and regulatory requirements - medical devices | Legal and regulatory requirements - medical devices no knowledge | Legal and regulatory requirements - medical devices basic knowledge | Legal and regulatory requirements - medical devices intermediate knowledge | Legal and regulatory requirements - medical devices advanced knowledge |
| Legal and regulatory requirements -  business and governance | Legal and regulatory requirements -  business and governance no knowledge | Legal and regulatory requirements -  business and governance basic knowledge | Legal and regulatory requirements -  business and governance intermediate knowledge | Legal and regulatory requirements -  business and governance advanced knowledge |
| Privacy requirements | Privacy requirements no knowledge | Privacy requirements basic knowledge | Privacy requirements intermediate knowledge | Privacy requirements advanced knowledge |
| Data security and ethics | Data security and ethics no knowledge | Data security and ethics basic knowledge | Data security and ethics intermediate knowledge | Data security and ethics advanced knowledge |
| Access to treatments and technologies | Access to treatments and technologies no knowledge | Access to treatments and technologies basic knowledge | Access to treatments and technologies intermediate knowledge | Access to treatments and technologies advanced knowledge |
| Societal impacts and policy development | Societal impacts and policy development no knowledge | Societal impacts and policy development basic knowledge | Societal impacts and policy development intermediate knowledge | Societal impacts and policy development advanced knowledge |
| Codes of conduct/compliance | Codes of conduct/compliance no knowledge | Codes of conduct/compliance basic knowledge | Codes of conduct/compliance intermediate knowledge | Codes of conduct/compliance advanced knowledge |

14. How do you rate your access to training/support on the following aspects of professional practice?

|  | no access | some access | moderate access | good access | excellent access |
| --- | --- | --- | --- | --- | --- |
| Medical ethics and genomics | Medical ethics and genomics no access | Medical ethics and genomics some access | Medical ethics and genomics moderate access | Medical ethics and genomics good access | Medical ethics and genomics excellent access |
| Communication of complex genomic information and variation by audience (HCP, peer, public) | Communication of complex genomic information and variation by audience (HCP, peer, public) no access | Communication of complex genomic information and variation by audience (HCP, peer, public) some access | Communication of complex genomic information and variation by audience (HCP, peer, public) moderate access | Communication of complex genomic information and variation by audience (HCP, peer, public) good access | Communication of complex genomic information and variation by audience (HCP, peer, public) excellent access |
| Inclusion and diversity as related to genomic disorders – minorities, disability, discrimination | Inclusion and diversity as related to genomic disorders – minorities, disability, discrimination no access | Inclusion and diversity as related to genomic disorders – minorities, disability, discrimination some access | Inclusion and diversity as related to genomic disorders – minorities, disability, discrimination moderate access | Inclusion and diversity as related to genomic disorders – minorities, disability, discrimination good access | Inclusion and diversity as related to genomic disorders – minorities, disability, discrimination excellent access |
| Collaboration with HCP or research groups | Collaboration with HCP or research groups no access | Collaboration with HCP or research groups some access | Collaboration with HCP or research groups moderate access | Collaboration with HCP or research groups good access | Collaboration with HCP or research groups excellent access |
| Collaboration with peers, patient advocacy groups, allied health practitioners | Collaboration with peers, patient advocacy groups, allied health practitioners no access | Collaboration with peers, patient advocacy groups, allied health practitioners some access | Collaboration with peers, patient advocacy groups, allied health practitioners moderate access | Collaboration with peers, patient advocacy groups, allied health practitioners good access | Collaboration with peers, patient advocacy groups, allied health practitioners excellent access |
| Collaboration with patients, patient support groups or public groups | Collaboration with patients, patient support groups or public groups no access | Collaboration with patients, patient support groups or public groups some access | Collaboration with patients, patient support groups or public groups moderate access | Collaboration with patients, patient support groups or public groups good access | Collaboration with patients, patient support groups or public groups excellent access |
| Continuous learning methodologies and impact on adaptability and innovation | Continuous learning methodologies and impact on adaptability and innovation no access | Continuous learning methodologies and impact on adaptability and innovation some access | Continuous learning methodologies and impact on adaptability and innovation moderate access | Continuous learning methodologies and impact on adaptability and innovation good access | Continuous learning methodologies and impact on adaptability and innovation excellent access |
| Leadership development in precision medicine | Leadership development in precision medicine no access | Leadership development in precision medicine some access | Leadership development in precision medicine moderate access | Leadership development in precision medicine good access | Leadership development in precision medicine excellent access |
| Entrepreneurship | Entrepreneurship no access | Entrepreneurship some access | Entrepreneurship moderate access | Entrepreneurship good access | Entrepreneurship excellent access |
| 21st century skills: critical thinking, creativity, problem-solving skills | 21st century skills: critical thinking, creativity, problem-solving skills no access | 21st century skills: critical thinking, creativity, problem-solving skills some access | 21st century skills: critical thinking, creativity, problem-solving skills moderate access | 21st century skills: critical thinking, creativity, problem-solving skills good access | 21st century skills: critical thinking, creativity, problem-solving skills excellent access |
| Project management skills | Project management skills no access | Project management skills some access | Project management skills moderate access | Project management skills good access | Project management skills excellent access |

15. Do you need additional training in precision medicine?

Yes

No

Uncertain

16. What type of training would you like in precision medicine?

Formal university degree

Formal university subject

Massive open online course (MOOC)

Short course delivered by industry organisation

Free online course

Paid online course

Courses provided by your employer

Podcasts

Your own reading/watching videos

Attending conferences

17. What type of new roles do you expect will be needed by industry in the future?

18. Looking to the future, what aspects of precision medicine do you anticipate that industry staff will need training on to do their job?
