# Supplementary material for: Building a Competency Framework to Integrate Inter-disciplinary Precision Medicine Capabilities into the Medical Technology and Pharmaceutical Industry
Source: Ther Innov Regul Sci. 2024 Mar 15;58(3):567–77. doi: 10.1007/s43441-024-00626-5 (PMC11043185; doi:10.1007/s43441-024-00626-5)
Supplement: Supplementary file 2 — Supplementary file2 (DOCX 51 KB) [file 43441_2024_626_MOESM2_ESM.docx]

Appendix 2: select survey responses

Current need for medical science and technology knowledge

*Need for medical science and technology knowledge in 5 years’ time*

*Current need for knowledge of translation and clinical application* *requirements*

*Need for translation and clinical application* *knowledge in 5 years’ time*

*Current need for knowledge of governance and regulation requirements*

*Need for governance and regulation knowledge in 5 years’ time*

*Current need for professional practice requirements*

*Need for professional practice skills in 5 years’ time*
